# Genetic variations in circadian rhythm genes and susceptibility for myocardial infarction

**DOI:** 10.1590/1678-4685-GMB-2017-0147

**Published:** 2018-05-14

**Authors:** Ivana Škrlec, Jakov Milic, Marija Heffer, Borut Peterlin, Jasenka Wagner

**Affiliations:** 1 University of Osijek University of Osijek Department of Medical Biology and Genetics Croatia Department of Medical Biology and Genetics, Faculty of Medicine, J. J. Strossmayer University of Osijek, Croatia; 2 University Medical Center Ljubljana University Medical Center Ljubljana Clinical Institute of Medical Genetics Slovenia Clinical Institute of Medical Genetics, University Medical Center Ljubljana, Slovenia; 3 University of Osijek University of Osijek Faculty of Dental Medicine and Health Croatia Faculty of Dental Medicine and Health, J. J. Strossmayer University of Osijek, Croatia

**Keywords:** cardiovascular diseases, circadian rhythm, myocardial infarction, polymorphisms

## Abstract

Disruption of endogenous circadian rhythms has been shown to increase the risk of developing myocardial infarction (MI), suggesting that circadian genes might play a role in determining disease susceptibility. We conducted a case-control study on 200 patients hospitalized due to MI and 200 healthy controls, investigating the association between MI and single nucleotide polymorphisms (SNPs) in four circadian genes (*ARNTL, CLOCK, CRY2,* and *PER2*). The variants of all four genes were chosen based on their previously reported association with cardiovascular risk factors, which have a major influence on the occurrence of myocardial infarction. Statistically significant differences, assessed through Chi-square analysis, were found in genotype distribution between cases and controls of the *PER2* gene rs35333999 (*p*=0.024) and the *CRY2* gene rs2292912 (*p*=0.028); the corresponding unadjusted odds ratios, also significant, were respectively OR=0.49 (95% CI 0.26-0.91) and OR=0.32 (95% CI 0.11-0.89). Our data suggest that genetic variability in the *CRY2* and *PER2* genes might be associated with myocardial infarction.

Cardiovascular diseases (CVD) are the world’s leading cause of death (WHO, 2015), and myocardial infarction (MI) is the third leading cause of mortality in Croatia (Hrabak Zerjavic *et al.*, 2010). In the last few years, genome-wide association studies (GWAS) have identified many genetic variants that contribute to a higher risk of MI (Erdmann *et al.*, 2010). Despite numerous studies conducted on MI, its etiology is still largely unknown.

Human physiological activities and diseases are under the control of circadian rhythm. Several physiological factors can cause MI, and several of these factors are known to oscillate with circadian rhythm (Kanth *et al.*, 2013). Some of those are blood pressure (Woon *et al.*, 2007; Englund *et al.*, 2009; Dashti *et al.*, 2014), glucose homeostasis (Dashti *et al.*, 2014; Lipkova *et al.*, 2014), vascular endothelial function, myocardial contractile function and metabolism (Ebisawa *et al.*, 2001; Martino and Sole, 2009; Bonney *et al.*, 2013a,b).

Epidemiological studies found daylight to be the leading regulator of the human circadian rhythm, and sunlight cycles are crucial for keeping a healthy cardiovascular system (Bonney *et al*., 2013a,b). In humans, relevant relationships exist between circadian clocks and the metabolic syndrome (Englund *et al.*, 2009).

There is increasing evidence that circadian rhythms have an important role in preserving homeostasis and appropriate body function including cardiac metabolism (Woon *et al.*, 2007; Englund *et al.*, 2009; Bonney *et al.*, 2013a,b; Corella *et al.*, 2016). In the mutant mouse models is emphasized that mutations in the *PER2* gene were associated with protection from myocardial ischemia (Bonney *et al.*, 2013a,b).

Circadian clock network consists of molecular components where *ARNTL*, *CLOCK, CRY2* and *PER2* genes represent the central node in the network (Corella *et al.*, 2016). Those core clock genes establish the internal clock and constitute negative and positive transcriptional and translational feedback loops. Heterodimers of the ARNTL/CLOCK proteins initiate the transcription of the *CRY2, PER2*, and other clock-related genes. Heterodimers of the CRY2/PER2 proteins assemble the negative feedback loop and inhibit the transcriptional activity of the *ARNTL* and *CLOCK* genes (Takeda and Maemura, 2010, 2016).

The aim of this study was to explore a possibility of association of the genetic variability of the *ARNTL*, *CLOCK, CRY2* and *PER2* genes with myocardial infarction in humans. We implemented a case-control study on a population of patients with myocardial infarction in comparison with a control population. The study was conducted from August 2012 to December 2013. Patients with myocardial infarction were hospitalized at the Clinical Department of Cardiovascular Diseases and Intensive Care at the University Hospital Osijek, Croatia.

Myocardial infarction was defined as the presence of at least two of the following: typical increase of biochemical marker of myocardial necrosis – cardiac troponin T (above the 99^th^ percentile), ischemic chest pain symptoms lasting more than 30 minutes, and electrocardiography changes (ECG) indicative of ischemia (ST-segment elevation or depression) (Thygesen *et al.*, 2012). Thirty-eight patients were excluded from this study because they did not meet the criteria as mentioned above or they had a percutaneous coronary intervention or coronary artery bypass grafting. Fifty-three patients refused to participate in the study, and nine patients drop out of the study.

The control group consisted of 200 healthy sex- and age-matched participants, whose medical documentation did not show any history of cardiovascular diseases. Their primary care physician chose them in an ambulatory. We excluded any patient’s relatives from the control group because of complex heritability of cardiovascular risk factors showed in monozygotic twins (Elder *et al.*, 2009).

Systematic information on the medical history was collected from all participants. The questionnaire included questions about age, history of smoking, hypertension, dyslipidemia, respiratory diseases, diabetes mellitus, kidney and liver diseases. All given information was checked in patient’s medical record.

This study was approved by the Ethics Committee of the Faculty of Medicine, the Josip Juraj Strossmayer University of Osijek (No. 2158-61-07-12-21) and by the Ethics Committee of the University Hospital Osijek (No. 25-1:3160-3/2012). The study was conducted according to the Declaration of Helsinki and its amendments. Written informed consent was obtained from all participants in the study.

In this study, genetic variants were genotyped in four key circadian rhythm regulating genes, *ARNTL*, *CLOCK*, *CRY2,* and *PER2*. Ten SNPs previously associated with cardiovascular risk factors were investigated. Six single nucleotide polymorphisms (SNPs) were chosen from *ARNTL* and *CLOCK* genes. Of these, three SNPs, rs3789327, rs4757144, and rs12363415 in the *ARNTL* gene, and three SNPs, rs11932595, rs6811520, and rs13124436 were selected in the *CLOCK* gene. Two SNPs, rs2292912 and rs10838524 in the *CRY2* gene, and two SNPs, rs35333999, and rs934945 were selected in the *PER2* gene.

Genomic DNA was extracted from the peripheral blood lymphocyte using standard procedures (QIAamp DNA Blood Mini Kit, Qiagen, Hilden, Germany). Genotyping was carried out by real-time PCR method performed on 7500 Real-Time PCR System (Applied Biosystems, Foster City, CA, USA) using TaqMan SNP genotyping assays. The PCR reaction mix of 6.25 μL final volume consisted of 6 ng of genomic DNA, 3.13 μL of TaqMan Universal PCR Master Mix 2X, 0.15 μL Assay Mix 40X, and 2.17 μL ddH_2_O. The protocol for PCR amplification was: initial denaturation step at 95 °C for 10 min, then 40 cycles of denaturation at 92 °C for 15 s, followed by 60 °C for 1 min, and a final extension at 60 °C for 1 min. The allelic discrimination analysis was performed using SDS 7500 Software Version 2.3 (Applied Biosystems, Foster City, CA, USA).

Chi-square tests (χ^2^) on contingency tables were used to compare allelic and genotype frequencies in controls and cases. To further assess the presence of associations, we calculated, for the indicated genetic risk factors, the odds ratios (OR) and their respective 95% confidence intervals (CI). Analyses were performed using SHEsis web tool (Shi and He, 2005; Li *et al.*, 2009). An additional level of genotyping quality control was performed using Chi-Square goodness-of-fit test, by comparing our genotype distribution with those predicted by Hardy-Weinberg equilibrium. Associations were considered significant when they reached the *p*-value of equal to or less than 0.05. Appropriate corrections of significance values were also applied using the Benjamini-Hochberg correction method (false-discovery rate – FDR values) because of multiple SNPs were investigated. The *q* values of less than 0.05 were considered to be significant. As the participants of the study were not related, haplotypes and the pairwise linkage disequilibrium (LD) were estimated using SNPStats web tool (Solé *et al.*, 2006).

The prevalence of cardiovascular risk factors among all participants included in the study sample is summarized in Table 1. The mean age of the study population was 64 ± 13 years, and 54.5% were males. Genotype frequencies of investigated polymorphisms were predicted by the Hardy-Weinberg equilibrium in the study and the control group (*p* > 0.05), except for rs6811520, which was excluded from further analyses. Minor allele frequencies of the almost all investigated SNPs are consistent with a reference population of HapMap phase 3, CEU; the exception was rs35333999 in the *PER2* gene and rs6811520 in the *CLOCK* gene (Table 2). Genotype and allelic distribution of the *ARNTL*, *CLOCK*, *CRY2* and *PER2* polymorphisms of the 200 MI patients and 200 healthy controls are shown in Table 2.

**Table 1:** Prevalence of cardiovascular risk factors among all participants included in the study by groups.

Variables	Patients (n=200)	Controls (n=200)	*p*-value
Age year mean (SD)	66 ± 12	62 ± 13	0.086
Gender: males	114 (57%)	104 (52%)	0.317
Smoking			
Non-smokers	99 (49.5%)	151 (75.5%)	< 0.001
Smokers	41 (20.5%)	39 (19.5%)	
Former smokers	60 (30%)	10 (5%)	
Hypertension	107 (53.5%)	59 (29.5%)	< 0.001
Dyslipidemia	26 (13%)	23 (11.5%)	0.648
Respiratory disease	2 (1.8%)	8 (4%)	0.055
Diabetes mellitus 2	44 (22%)	0	-
Kidney diseases	15 (7.5%)	12 (6%)	0.550
Liver disease	10 (5%)	9 (4.5%)	0.814

Numerical variables are presented as mean (SD), while categorical variables as number (percentage).

**Table 2:** Allele and genotype distribution and frequencies of the *ARNTL*, *CLOCK*, *CRY2* and *PER2* polymorphisms

Gene	SNP	Minor allele	MAF* patients	MAF* controls	*p*-value	Genotype	Genotype frequency (%)				
							Patients with MI	Controls	*p*-value	X^2^	q value
*ARNTL1*	rs3789327	C	0.42	0.44	0.475	CC	33.5	34.0	0.301	2.34	0.443
						CT	50.0	44.0			
						TT	16.5	22.0			
	rs4757144	A	0.62	0.59	0.385	AA	39.5	36.0	0.694	0.73	0.867
						AG	45.5	46.5			
						GG	15.0	17.5			
	rs12363415	G	0.15	0.17	0.501	AA	71.0	71.0	0.120	4.24	0.300
						AG	27.5	24.0			
						GG	1.5	5.0			
*CLOCK*	rs11932595	G	0.39	0.39	0.942	AA	36.5	35.0	0.878	0.26	0.878
						AG	49.5	52.0			
						GG	14.0	13.0			
	rs6811520	T	0.39	0.49	0.004	CC	37.5	31.0	0.005	10.55	0.050
						CT	47.0	40.0			
						TT	15.5	29.0			
	rs13124436	A	0.33	0.28	0.143	AA	11.0	7.0	0.294	2.45	0.443
						AG	43.0	41.5			
						GG	46.0	51.5			
*CRY2*	rs2292912	G	0.20	0.26	0.054	CC	62.0	55.5	0.056	5.78	0.197
						CG	35.5	37.0			
						GG	2.5	7.5			
	rs10838524	A	0.45	0.49	0.230	AA	19.0	26.3	0.212	3.11	0.424
						AG	52.5	46.5			
						GG	28.5	27.2			
*PER2*	rs35333999	T	0.05	0.09	0.032	CC	91.5	84.0	0.059	5.64	0.197
						CT	7.5	15.0			
						TT	1.0	1.0			
	rs934945	T	0.17	0.19	0.644	CC	69.0	67.5	0.864	0.29	0.878
						CT	27.5	28.0			
						TT	3.5	4.5			

* MAF –*minor allele frequency*

Pearson Chi-square test

*CLOCK* SNP rs6811520 showed a departure from the Hardy-Weinberg equilibrium and was excluded

*p*-values shown in the table are corrected for the multiple comparisons.

We did not find any significant associations between rs3789327, rs4757144, and rs12363415 in the *ARNTL* gene and MI, and there was no significant difference comparing the frequencies of four the most frequent haplotypes for the three analyzed SNPs in the *ARNTL* gene in the patients and control groups.

The SNPs in the *CLOCK* gene, rs11932595, and rs13124436 did not show significant association of genotype or allelic distribution between the patients and control groups. Accordingly, we did not find any significant difference when comparing the frequencies of four most frequent haplotypes for the two analyzed SNPs in the *CLOCK* gene in the study and control groups.

No significant associations were found between the rs2292912 and rs10838524 SNPs of the *CRY2* gene and MI. Under the recessive genotype model (GG versus CC + CG), OR of 0.32 was estimated for the *CRY2* gene polymorphism rs2292912 (*p*=0.028, OR=0.32 with 95% CI 0.11-0.89) (Table 3). We did not find any significant difference when comparing the frequencies of the three most frequent haplotypes for the two analyzed SNPs in the *CRY2* gene in the patients and control groups.

**Table 3:** Genotype models of the *ARNTL*, *CLOCK*, *CRY2* and *PER2* polymorphisms.

	Dominant model			Recessive model			Codominant model		
*ARNTL1*	*p*	OR	95% CI	*p*	OR	95% CI	*p*	OR	95% CI
rs3789327	0.916	0.98	0.65-1.48	0.163	0.70	0.42-1.16	0.229	0.79	0.53-1.17
rs4757144	0.498	0.83	0.49-1.42	0.470	1.16	0.77-1.74	0.841	1.04	0.7-1.54
rs12363415	0.780	1.07	0.69-1.66	0.040	3.53	0.95-13.14	0.423	0.83	0.53-1.31
*CLOCK*									
rs11932595	0.770	1.09	0.61-1.95	0.754	1.07	0.71-1.61	0.617	1.10	0.75-1.64
rs13124436	0.271	0.80	0.54-1.19	0.162	1.63	0.81-3.38	0.761	0.94	0.63-1.40
*CRY2*									
rs2292912	0.187	0.76	0.51-1.14	0.028	0.32	0.11-0.89	0.051	0.35	0.12-1.01
rs10838524	0.785	0.94	0.61-1.46	0.084	0.66	0.41-1.06	0.082	0.64	0.39-1.06
*PER2*									
rs35333999	0.024	0.49	0.26-0.91	1	1	0.14-7.17	0.509	0.50	0.06-3.91
rs934945	0.747	0.93	0.61-1.42	0.611	0.77	0.28-2.11	0.665	0.79	0.28-2.27

*p*-values shown in the table are corrected for the multiple comparisons.

A statistically significant difference was seen in the allelic distribution of rs35333999 (*p*=0.033) in the *PER2* gene. However, we did not find any significant association between the rs934945 polymorphism of the *PER2* gene and MI. Under the dominant genotype model (TT + CT versus CC), an OR of 0.49 was estimated for the *PER2* gene polymorphism rs35333999 (*p*=0.024, OR=0.49 with 95% CI 0.26-0.91) (Table 3).

We analyzed the completed haplotypes in the four investigated genes. Table 4 shows the frequencies of predicted haplotypes in the patients and the control group. A statistically significant difference in haplotype distributions was confirmed at the *PER2* gene locus when comparing the frequency of haplotype TC (*p*=0.033) between participants with MI and control group.

**Table 4:** Frequencies and distribution of probable haplotypes in the patients and the control groups.

Gene	rs3789327	rs4757144	rs12363415	Frequency patients	Frequency controls	*p*-value
*ARNTL*	T	G	A	0.16	0.19	0.562
*ARNTL*	C	A	A	0.16	0.18	0.604
*ARNTL*	C	G	A	0.13	0.13	0.627
*ARNTL*	T	A	A	0.39	0.35	0.073
*CLOCK*	rs11932595	rs13124436				
*CLOCK*	A	G		0.36	0.39	0.381
*CLOCK*	G	G		0.32	0.33	0.820
*CLOCK*	A	A		0.25	0.21	0.276
*CLOCK*	G	A		0.07	0.06	0.776
*CRY2*	rs2292912	rs10838524				
*CRY2*	C	A		0.25	0.24	0.616
*CRY2*	C	G		0.54	0.50	0.232
*CRY2*	G	A		0.19	0.26	0.052
*PER2*	rs35333999	rs934945				
*PER2*	C	C		0.78	0.73	0.101
*PER2*	C	T		0.17	0.19	0.646
*PER2*	T	C		0.05	0.09	0.033

Linkage disequilibrium calculated for the *CLOCK* gene SNPs (rs11932595 and rs13124436) were D’=0.06, r^2^=0.001. SNPs in the *CRY2* gene were in LD (D’=0.97, r^2^=0.31). There was no LD between SNPs in the *ARNTL* gene, LD between rs4757144 and rs12363415 was D’=0.15, r^2^=0.001, between rs3789327 and rs4757144 was D’=0.10, r^2^=0.01, and between rs3789327 and rs12363415 was D’=0.61, r^2^=0.09. LD for SNPs in the *PER2* gene (rs35333999 and rs934945) was D’=0.001, r^2^=0.01.

In this case-control study we found an association between MI and gene variants of the *CRY2* and *PER2* gene in a sample of 400 participants. The circadian clock is a 24-hour internal system that allows an organism to maintain environmental changes and acclimate to them. Therefore, circadian rhythms handle a broad diversity of physiological and metabolic functions, and any interruption of these rhythms may influence on human health.

Two feedback loops, ARNTL/CLOCK and CRY/PER control expression of downstream transcription factors which regulate downstream target genes involved in different biochemical pathways, such as metabolism of glucose and lipids, synthesis of cholesterol, and others (Staels, 2006). A small number of studies have considered the role of the circadian rhythm in MI. One suggested that gene expression of the cardiomyocyte circadian clock influences myocardial contractile function, metabolism and gene expression (Bray *et al.*, 2008). Another showed that in the cardiomyocyte-specific circadian clock mutant mice, the clock is a direct regulator of triglyceride metabolism in the heart (Tsai *et al.*, 2010), while deletion of *ARNTL* in mice adipocyte resuled in obesity (Paschos *et al.*, 2012).

*PER2* is involved in the regulation of fatty acid metabolism with increased oxygen consumption (Grimaldi *et al.*, 2010). Lipolysis was markedly attenuate in circadian clock mutant mice hearts, and there is a potential explanation for accelerated metabolic pathologies, such as atherosclerosis which might lead to MI in patients (Tsai *et al.*, 2010). *PER2* knock-out mice had larger infarct sizes, and the cardiac *PER2* have an important role in fatty acid metabolism and inflammation during myocardial ischemia and reperfusion (Bonney *et al.*, 2013b).

Depletion of glycogen stores leads to increased infarct sizes in *PER2* mutated mice because of reduced glycolysis during myocardial ischemia (Eckle *et al.*, 2012). It has been shown that the protein PER2 has a cardioprotective role during myocardial ischemia in mice (Bonney *et al.*, 2013a), and mutation of the *PER2* gene is associated with a shorter circadian period during constant darkness (Vukolic *et al.*, 2010). The study of Suarez-Barrientos *et al.* (2011) found that infarct size was larger in the early morning, what is similar to the findings that light-dependent stabilization of *PER2* had cardioprotection role in ischemia (Eckle *et al.*, 2012).

Genetic variations in the *PER2* gene are associated with abdominal obesity (Garaulet *et al.*, 2010) and metabolic syndrome (Garcia-Rios *et al.*, 2012) due to its part in the lipid metabolism. *PER2* activation during ischemia regulates fatty acid beta-oxidation during ischemia and inflammation during reperfusion by increasing inflammatory cytokines, metabolism and inflammation are connected, and inflammation can be a consequence of pathologic metabolism (Bonney *et al.*, 2013b). Thereby, patients who have metabolic syndrome and higher inflammatory markers are at greater risk to develop CVD (Haffner, 2006). Although the genetic variation rs35333999 in the *PER2* gene and rs2292912 in the *CRY2* gene were associated with MI in this study, they are not precise because of the broad 95% CI values.

Disruption of the circadian clock has been implicated in the pathogenesis of cardiovascular disease, for which hypertension is a major factor (Kovanen *et al.*, 2015). Aortic endothelial dysfunction with decreased production of nitric oxide was found in the mice with the mutated *PER2* gene, as well as, decreased vasodilatory prostaglandins and elevated the release of cyclooxygenase-1-derived vasoconstrictors (Scott, 2015). In endothelial hemostatic function *CLOCK*, thrombomodulin, and plasminogen activator inhibitor-1 are involved. A circadian clock controls those genes in endothelial cells (Scott, 2015), and disruption of those genes might lead to atherosclerosis and MI. Some genetic variants of the *CLOCK* gene are related to obesity (Bandín *et al.*, 2013; Garcia-Rios *et al.*, 2012), metabolic syndrome, and CVD (Garcia-Rios *et al.*, 2012).

The links from genetic variants to physiologic functions are most likely less than predicted, a study identified increased weight and obesity and features of metabolic syndrome as characteristics of *CLOCK*-deficient mice (Turek *et al.*, 2005). Studies on *CLOCK* mutant mouse indicate an important role of myocardial *CLOCK* gene in energy metabolism, myocardial contractility, and in the diurnal heart rate control (Scott, 2015). In response to a high-fat diet, mutations in *BMAL1* and *CLOCK* genes adjust circadian variation in glucose and triglycerides levels and affect the progress of insulin resistance (Scott, 2015).

Circadian rhythm has a significant role in regulating glucose metabolism, and cryptochromes are critical components of the circadian system in regulating glucose homeostasis (Kelly *et al.*, 2012; Lipkova *et al.*, 2014), dysregulation of which can lead to diabetes mellitus type 2. Genetic variations of the *BMAL1* gene, the mouse analog of the human *ARNTL* gene, are associated with diabetes mellitus type 2 and hypertension, providing evidence for the role of *ARNTL* variants in the pathology of the metabolic syndrome in human (Woon *et al.*, 2007).

Human studies have identified genetic variants and expression patterns of circadian clock genes, such as *ARNTL*, *CLOCK*, *CRY2*, *NPAS2* or *PER2*, that are associated with metabolic syndrome, hypertension or diabetes mellitus type 2 (Ohkura *et al.*, 2006; Scott, 2015). Circadian clock genes play a major role in hemostatic balance by regulating the fibrinolytic systems, and *CLOCK* and *CRY* genes are directly involved in this activity (Ohkura *et al.*, 2006), and therefore increase the risk for CVD. A role of the circadian rhythm in cardiovascular function is firmly supported in all those studies, but our study found the connection of myocardial infarction and some of the circadian rhythm genes SNPs.

Although some of the obtained results are significant, they are hardly suitable for diagnosis and prognosis purposes. A limitation of our study is its sample size. The sample size was relatively small and could yield false positive results. Furthermore, the high ORs and broad 95% CIs, as well as the low frequency of some genotypes does not allow adjusting for clinical and demographic confounders (i.e., multiple regression analysis), and low frequency of some genotypes may have resulted in insufficient statistical power to detect a positive association. For participants in control groups, there is a risk of developing some of the CVD.

In conclusion, we provide data indicating that genetic variability in the *CRY2* and *PER2* genes may be associated with MI. This suggests a role for the circadian rhythm in the development of myocardial infarction, but genetic variations in *ARNTL* and *CLOCK* genes are not directly associated with MI. Further verification and mechanistic analysis of the circadian system in MI are possible.
